# Selective microstructural integrity impairments of the anterior corpus callosum are associated with cognitive deficits in obstructive sleep apnea

**DOI:** 10.1002/brb3.1482

**Published:** 2019-11-20

**Authors:** Biao Zhang, Dao‐min Zhu, Wenming Zhao, Yu Zhang, Ying Yang, Cun Zhang, Jiajia Zhu, Yongqiang Yu

**Affiliations:** ^1^ Department of Radiology The First Affiliated Hospital of Anhui Medical University Hefei China; ^2^ Department of Sleep Disorders Affiliated Psychological Hospital of Anhui Medical University Hefei China; ^3^ Hefei Fourth People's Hospital Hefei China; ^4^ Anhui Mental Health Center Hefei China

**Keywords:** cognitive deficits, corpus callosum, diffusion tensor imaging, obstructive sleep apnea, subregion, tractography

## Abstract

**Background:**

There is some evidence that obstructive sleep apnea (OSA) patients have white matter integrity abnormality in the corpus callosum (CC). However, whether the CC subregions are differentially affected in OSA is largely unknown.

**Methods:**

Twenty patients with OSA and 24 well‐matched healthy controls were enrolled and underwent diffusion tensor imaging (DTI) and clinical and cognitive assessments. DTI tractography was used to reconstruct the CC which was divided into five subregions. Intergroup differences in multiple diffusion metrics of each CC subregion and their correlations with clinical and cognitive parameters were tested.

**Results:**

In comparison with healthy controls, OSA patients exhibited white matter integrity alterations in the anterior CC, characterized by increased radial diffusivity (RD) in the subregion 1 and decreased fractional anisotropy (FA) along with increased mean diffusivity (MD) and RD in the subregion 2. Moreover, we found that the lower microstructural integrity in the anterior CC was correlated with worse prospective memory and sustained attention in OSA patients.

**Conclusions:**

These findings indicate that the selective impairments of the anterior CC may help clarify the neural correlates of cognitive impairments in OSA.

## Graphical abstract

These findings suggest that the selective impairments of the anterior corpus callosum may help clarify the neural correlates of cognitive deficits in obstructive sleep apnea.

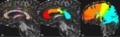

## 1 INTRODUCTION

Obstructive sleep apnea (OSA) is a chronic disorder characterized by frequent events of complete or partial obstruction of the upper airway during sleep, resulting in intermittent nocturnal hypoxia and sleep fragmentation (Young, Peppard, & Gottlieb, 2002). There is evidence that OSA patients have extensive cognitive impairments including memory and attention, which may be attributable to brain impairments caused by sleep disruption and blood gas abnormalities (Lal, Strange, & Bachman, 2012; Olaithe, Bucks, Hillman, & Eastwood, 2018; Vaessen, Overeem, & Sitskoorn, 2015). Despite considerable variation in the findings, both global and regional white matter microstructural alterations in OSA patients have been revealed by using diffusion tensor imaging (DTI) technique (Castronovo et al., 2014; Chen et al., 2015; Kumar et al., 2012, 2014; Macey et al., 2008; Macey, Kumar, Yan‐Go, Woo, & Harper, 2012; Xiong et al., 2017). Among these regional alterations, the corpus callosum (CC) damage is the most frequently and consistently reported white matter change in OSA (Kumar et al., 2012, 2014; Macey et al., 2008, 2012; Xiong et al., 2017). Given the vital role of the CC in interhemispheric communication linked to cognitive functioning (Doron & Gazzaniga, 2008), it is reasonable to assume that the CC microstructural disruption is a characteristic brain impairment of OSA, which may be responsible for cognitive impairments in this disorder.

Diffusion tensor imaging can be employed to measure white matter microstructural integrity by characterizing directional properties of the water diffusion. Fractional anisotropy (FA), mean diffusivity (MD), axial diffusivity (AD), and radial diffusivity (RD) are the four commonly used parameters of DTI (Alexander et al., 2011). FA and MD are summary measures which reflect the coherence of fiber tracts and the average rate of water diffusion, respectively. AD and RD are more directionally specific measures which reflect axonal and myelin integrity, respectively (Budde, Xie, Cross, & Song, 2009; Kumar et al., 2011; Song et al., 2002, 2003, 2005). Although voxel‐wise analyses of these diffusion metrics allow a fully automated investigation of white matter integrity in the whole brain, they have some problems in diffusion image spatial normalization and multiple comparison correction. On the contrary, DTI‐based fiber tractography can in vivo reconstruct white matter fibers between distant brain regions, which may facilitate a detection of microstructural impairments in specific fiber tracts (Basser, Pajevic, Pierpaoli, Duda, & Aldroubi, 2000; Conturo et al., 1999; Kanaan et al., 2006). CC can be divided into five segments according to the specific brain areas that these segments connect (Hofer & Frahm, 2006). Exploring CC changes at the subregional level by means of the tractographic method can help in understanding the role of CC in many neuropsychiatric conditions including major depressive disorder (Yamada et al., 2015), bipolar disorder (Yamada et al., 2015), alcohol dependence (Liu et al., 2010; Wang et al., 2019), and post‐traumatic stress disorder (Zhang et al., 2015). However, whether the CC subregions are differentially affected in OSA is still unclear.

In this study, our aims were (a) to investigate the microstructural integrity change pattern of each CC subregion in OSA patients by using tractography and multiple diffusion metrics, and (b) to test the correlations between CC alterations and clinical and cognitive parameters in OSA patients.

## 2 METHODS

### 2.1 Participants

This study included a total of 44 right‐handed subjects, including 20 OSA patients and 24 gender‐, age‐, and education‐matched healthy controls. Moderate‐to‐severe OSA patients (i.e., apnea–hypopnea index [AHI] >15) were enrolled from the Department of Sleep Disorders of Hefei Fourth People's Hospital. Controls were recruited from the local community through poster advertisements and screened by a detailed interview to ensure an absence of symptoms including chronic snoring or nocturnal apneas. For both patients and controls, exclusion criteria were as follows: (a) clinical diagnosis of sleep or respiratory disorders other than OSA; (b) the presence of severe psychiatric, neurologic or physical diseases that would affect the study results; (c) a lifetime history of drug abuse or dependence, or current intake of psychoactive medications; and (d) MRI contraindications such as claustrophobia or ferromagnetic implants. This study was approved by the Ethics Committee of The First Affiliated Hospital of Anhui Medical University. Written informed consent was obtained from all participants before the study procedure.

### 2.2 Polysomnography

Obstructive sleep apnea patients underwent overnight polysomnography examinations on an Embla N7000 instrument. Based on the American Academy of Sleep Medicine guidelines, an obstructive apnea was defined as a decrease in airflow ≥90% lasting at least 10 s and associated with persistent respiratory effort; a hypopnea was defined as a decrease in airflow ≥30% lasting at least 10 s and accompanied with a 4% or greater oxygen desaturation, or with EEG arousal. AHI was defined as the number of apnea and hypopnea events per hour of sleep. Patients with an AHI of 5–≥15, >15–30, and >30 were considered to have mild, moderate, and severe OSA, respectively. The mean and lowest nocturnal oxygen saturation (SaO_2_) values were also recorded. Before the polysomnography examination, excessive daytime sleepiness of each subject was assessed by the Epworth Sleepiness Scale (ESS; Johns, 1991).

### 2.3 Cognition assessment

The cognitive tests for prospective memory (PM) and sustained attention were conducted by an experienced psychiatrist. PM is defined as the ability to remember to conduct an intended action after a delay without any clues to do so (Einstein & McDaniel, 1990; McDaniel & Einstein, 2011). PM is typically classified into event‐based prospective memory (EBPM; i.e., the ability to remember to conduct an intended action at the occurrence of a certain event) and time‐based prospective memory (TBPM; i.e., the ability to remember to conduct an intended action at a certain time). Previous literature has described the EBPM and TBPM tests in detail (Yang, Zhong, Qiu, Cheng, & Wang, 2015; Zhu et al., 2019). For the EBPM test, the experimental stimuli were 30 cards in total, each comprising 12 Chinese words, of which 10 belonged to a major category and the other two belonged to a minor one. Participants were asked to read out the two words belonging to the minor category on each card. If the selected words belonged to the category of animals (target events), participants were required to tap the table. When the test was completed, participants should write down their telephone number without any clues. There were six target cards (card numbers 5, 10, 15, 20, 24, and 29) in this test. Participants received one point for each correct response to a target card and received two points for remembering to write down their phone number. The maximum score was 8 points. For the TBPM test, the experimental stimuli were 100 cards in total, each comprising 12 two‐digit numbers. Participants were asked to pick out the smallest and the largest numbers on each card and to tap the table at the target time points (5, 10, and 15 min after the start of the test). The test stopped at the 17‐min time point. Participants received two points if they responded within 10 s around the target time, and one point if within 30 s. The maximum score was 6 points.

Sustained attention was measured by using a computerized version of the Continuous Performance Task‐Identical Pairs (CPT‐IP; Cornblatt, Risch, Faris, Friedman, & Erlenmeyer‐Kimling, 1988). The stimuli were 2‐, 3‐, or 4‐digit numbers in separate conditions, which resulted in separate scores reflecting increasing memory load on digit span. Participants were instructed to monitor numbers on a computer screen and respond to any consecutive presentation of identical stimuli by key pressing as quickly as possible. Responses to target trials (pairs that were identical and required a response) and catch trials (pairs that were similar but not identical) were scored as true‐ and false‐positive responses, respectively. The main outcome parameter of interest is *d*′—a discrimination sensitivity index incorporating both true‐ and false‐positive responses. CPT‐IP‐2, CPT‐IP‐3, and CPT‐IP‐4 represented *d*′ values corresponding to the number of digits.

### 2.4 DTI data acquisition

Diffusion tensor imaging data were obtained using an 24‐channel head coil on a 3T MRI scanner (Discovery MR750w; General Electric) in The First Affiliated Hospital of Anhui Medical University. Foam pads and earplugs were used to limit head motion and minimize scanner noise. All subjects were instructed to move as little as possible during the scans. DTI data were acquired by a spin‐echo single‐shot echo planar imaging (SE‐SS‐EPI) sequence with the following parameters: repetition time = 10,000 ms; echo time = 74 ms; field of view = 256 mm × 256 mm; matrix = 128 × 128; slice thickness = 3 mm without gap; 50 axial slices; 64 diffusion gradient directions (*b* = 1,000 s/mm^2^) plus five *b* = 0 reference images; and acquisition time = 700 s. None of the participants were excluded for visually inspected imaging artifacts.

### 2.5 DTI data preprocessing and whole‐brain fiber tracking

The DTI preprocessing was performed by using the FMRIB Software Library (FSL, http://www.fmrib.ox.ac.uk/fsl) (Smith et al., 2004), Diffusion Toolkit (DTK, http://trackvis.org/dtk) and Pipeline for Analyzing brain Diffusion images (PANDA, http://www.nitrc.org/projects/panda; Cui, Zhong, Xu, He, & Gong, 2013). Specifically, the diffusion‐weighted images were first registered to the first b0 image by using affine transformations to minimize image distortions due to the eddy currents and head motions. After skull‐stripping, the 6 independent components of the diffusion tensor were computed. Then, FA, MD, AD, and RD were derived from on the diffusion tensor. Then, a deterministic streamline tracking algorithm, that is, Fiber Assignment by Continuous Tracking (FACT), was used to conduct the whole‐brain fiber tractography (Mori, Crain, Chacko, & van Zijl, 1999) with the FA threshold of 0.2 and the maximum curvature angle of 45°.

### 2.6 Fiber tracking of the corpus callosum

**Figure 1:**
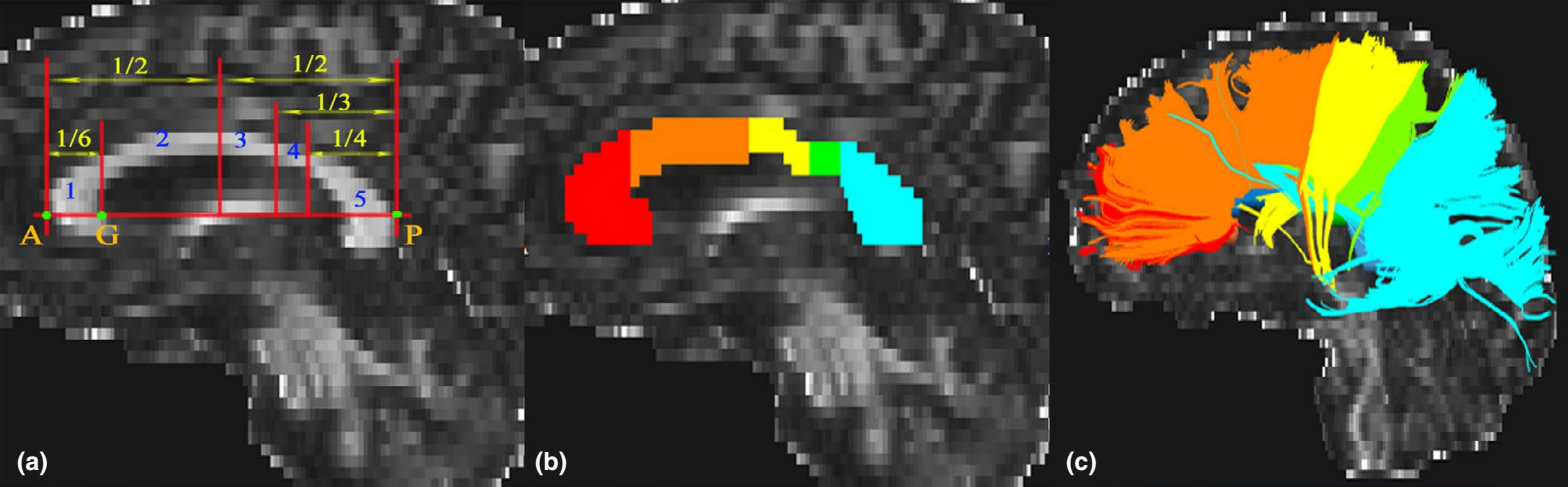
Subregions of the corpus callosum. Segmentation scheme of the corpus callosum (a and b). A and P are the anteriormost and posteriormost points, respectively. G is the anteriormost point on the inner convexity of the anterior callosum. A–P was used as the primary axis, lines perpendicular to which subdivide the corpus callosum into five subregions. Fibers crossing through each subregion on sagittal anatomical images (c)

Five subregions of the CC were established based on a previous topographical parcellation scheme that was selected because of its derivation of callosal boundaries from human studies (Hofer & Frahm, 2006). The cortical projections of the five subregions are as follows: subregion 1, prefrontal cortex; subregion 2, premotor and supplementary motor cortex; subregion 3, motor cortex; subregion 4, sensory cortex; and subregion 5, parietal, temporal, and occipital cortex (Figure 1a). Two trained raters who were blind to subjects' information manually divided each CC into subregions on the mid‐sagittal section of the FA maps using TrackVis software (http://www.trackvis.org) (Figure 1b). Then, the whole CC and five subregions were tracked separately (Figure 1c) and the average FA, MD, AD, RD, and of the 6 fibers were extracted for each participant. The intra‐class correlation coefficients (ICC) of inter‐rater measures ranged from 0.85 to 1, implying an excellent inter‐rater reliability (Table S1). The average values of the two raters' manual measurements were used for further statistical analyses.

### 2.7 Statistical analysis

Statistical analysis was conducted using the SPSS 23.0 software package (SPSS). Age, years of education, body mass index (BMI), clinical, and cognitive parameters were compared between OSA patients and healthy controls by using two‐sample *t* tests. Intergroup difference in gender was tested by using Pearson chi‐square test. General linear model was used to identify FA, MD, AD, and RD of the five subregions and whole CC that exhibited intergroup differences after controlling for BMI. Moreover, Pearson's correlation coefficients were measured to investigate the associations between diffusion metrics exhibiting significant intergroup differences and clinical and cognitive parameters (i.e., AHI, Mean SaO_2_, Nadir SaO_2_, ESS, EBPM, TBPM, and CPT‐IP) in the patient group. For all these statistical analyses, *p* < .05 was considered to indicate statistical significance because of the exploratory nature of this study.

## 3 RESULTS

### 3.1 Subjects' characteristics

Table 1 shows demographic, clinical, and cognitive data of the sample. In brief, the patient and control groups had no significant differences in gender (chi‐square test, *χ* ^2^ = 1.605, *p* = .205), age (two‐sample *t* test, *t* = 0.768, *p* = .447), education (*t* = 0.301, *p* = .765), EBPM (*t* = −1.210, *p* = .233), TBPM (*t* = −0.920, *p* = .363), CPT‐IP‐2 (*t* = 1.047, *p* = .301), CPT‐IP‐3 (*t* = −0.264, *p* = .793), and CPT‐IP‐4 (*t* = 0.939, *p* = .353). The higher proportion of male patients in the current cohort reflects the higher prevalence of OSA among males in the general population (Young, Evans, Finn, & Palta, 1997). As expected, OSA patients had a significantly higher score for BMI (*t* = 4.591, *p* < .001) and ESS (*t* = 5.509, *p* < .001) compared with healthy controls.

**Table 1:** Demographic, clinical, and cognitive characteristics

Characteristics	OSA (*n* = 20)	HC (*n* = 24)	Statistics	*p* Value
Gender (female/male)	16/4	15/9	*χ* ^2^ = 1.605	.205^a^
Age (years)	43.1 ± 10.5	40.7 ± 10.0	*t* = 0.768	.447^b^
Education (years)	12.5 ± 4.4	12.1 ± 3.8	*t* = 0.301	.765^b^
BMI (kg/m^2^)	26.3 ± 2.5	22.8 ± 2.4	*t* = 4.591	<.001^b^
AHI	49.0 ± 22.5	NA	NA	NA
Mean SaO_2_ (%)	94.1 ± 2.4	NA	NA	NA
Nadir SaO_2_ (%)	77.9 ± 8.3	NA	NA	NA
ESS	9.3 ± 5.0	2.9 ± 2.4	*t* = 5.509	<.001^b^
EBPM	4.2 ± 2.9	5.2 ± 2.9	*t* = −1.210	.233^b^
TBPM	3.2 ± 2.1	3.8 ± 1.9	*t* = −0.920	.363^b^
CPT‐IP‐2	3.1 ± 0.8	2.8 ± 1.1	*t* = 1.047	.301^b^
CPT‐IP‐3	2.5 ± 0.8	2.6 ± 0.9	*t* = −0.264	.793^b^
CPT‐IP‐4	1.7 ± 0.7	1.4 ± 0.9	*t* = 0.939	.353^b^

Note

The data are presented as the mean ± standard deviation.

Abbreviations: AHI, apnea/hypopnea index; BMI, body mass index; CPT‐IP, Continuous Performance Task‐Identical Pairs; EBPM, event‐based prospective memory; ESS, Epworth sleepiness scale; HC, healthy controls; NA, not available; OSA, obstructive sleep apnea; SaO_2_, oxygen saturation; TBPM, time‐based prospective memory.

a The *p* value was obtained by Pearson chi‐square test.

b The *p* values were obtained by two‐sample *t* tests.

### 3.2 Group differences in diffusion metrics of the corpus callosum

**Figure 2:**
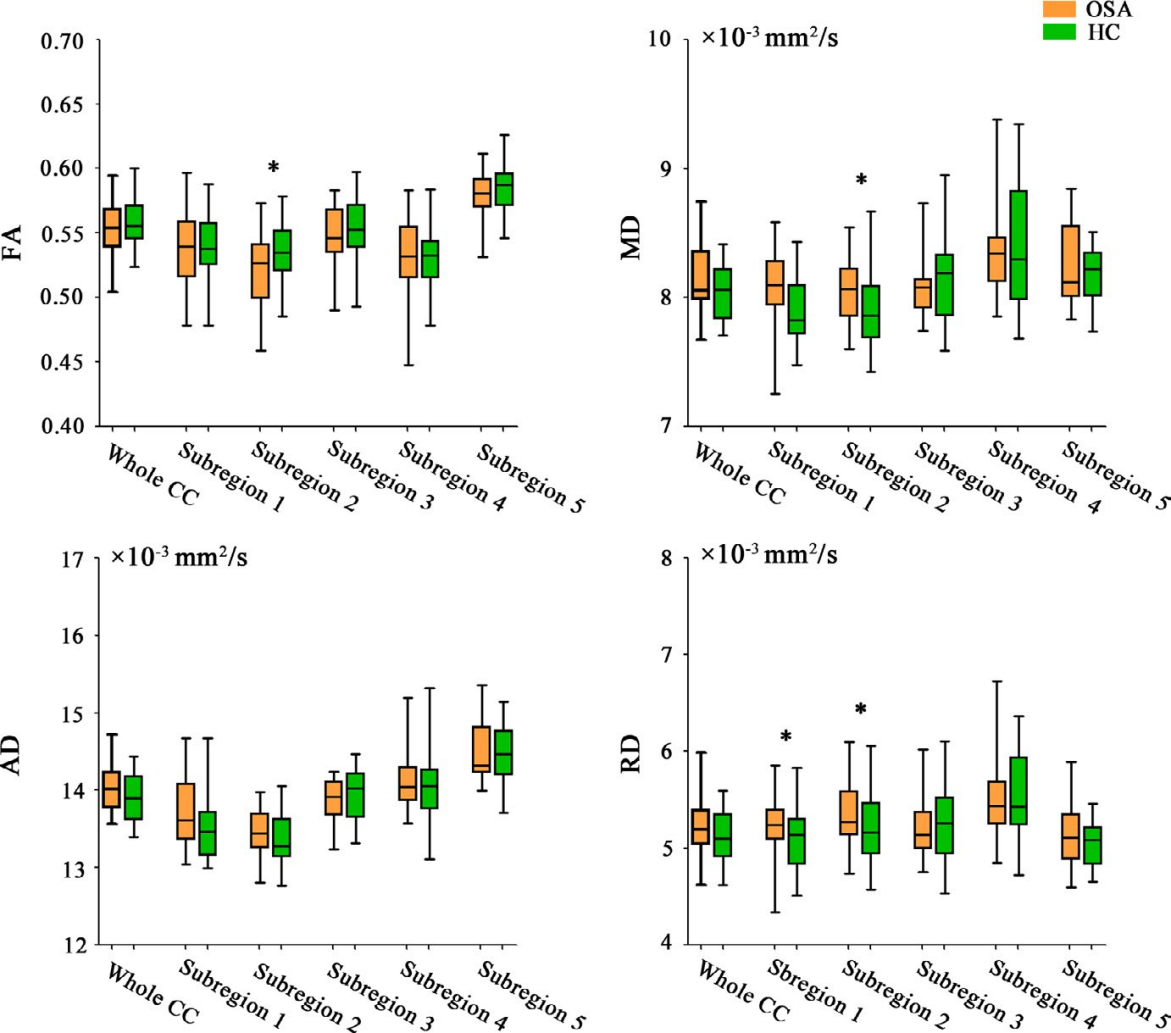
Alterations in diffusion metrics of the corpus callosum subregions. The horizontal line in the middle of each box indicates the median, while the top and bottom borders mark the 75th and 25th percentiles, respectively. The whiskers above and below the box mark the maximum and minimum values. Abbreviations: AD, axial diffusivity; CC, corpus callosum; FA, fractional anisotropy; HC, healthy controls; MD, mean diffusivity; OSA, obstructive sleep apnea; RD, radial diffusivity

The group differences in FA, MD, AD, and RD of the callosal subregions and whole CC between OSA patients and healthy controls are illustrated in Figure 2, Tables S2 and S3. Specifically, patients exhibited significantly increased RD (*F* = 4.518, *p* = .040) in the subregion 1 relative to controls. We also found significantly reduced FA (*F* = 4.882, *p* = .033) as well as increased MD (*F* = 5.042, *p* = .030) and RD (*F* = 5.742, *p* = .021) of the subregion 2 in OSA patients. However, no significant differences in any diffusion metrics of the subregions 3–5 and whole CC were observed.

### 3.3 Associations of the callosal integrity changes with clinical and cognitive variables

**Figure 3:**
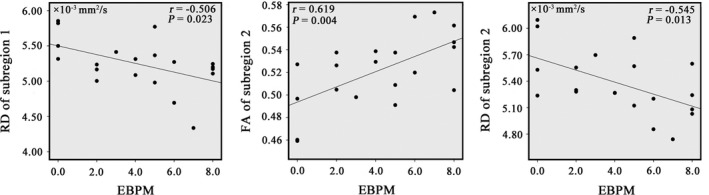
Correlations between diffusion metrics of the corpus callosum subregions and event‐based prospective memory in patients with obstructive sleep apnea. Abbreviations: EBPM, event‐based prospective memory; FA, fractional anisotropy; RD, radial diffusivity

**Figure 4:**
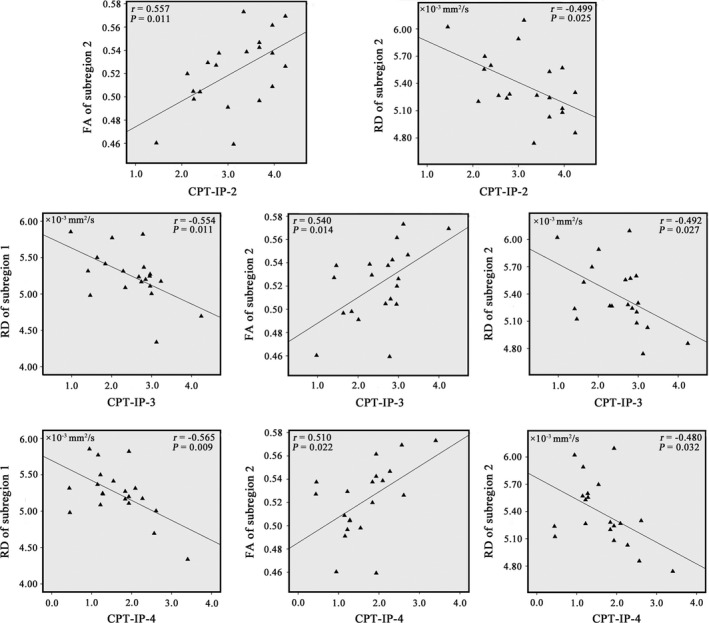
Correlations between diffusion metrics of the corpus callosum subregions and CPT‐IP in patients with obstructive sleep apnea. Abbreviations: CC, corpus callosum; CPT‐IP, Continuous Performance Task‐Identical Pairs; FA, fractional anisotropy; RD, radial diffusivity

As shown in Figure 3, EBPM was negatively correlated with RD of the subregion 1 (*r* = −.506, *p* = .023) and subregion 2 (*r* = −.545, *p* = .013), and positively correlated with FA of the subregion 2 (*r* = .619, *p* = .004) in the patient group. The correlations between diffusion metrics of the corpus callosum and CPT‐IP in the patients are illustrated in Figure 4. Specifically, CPT‐IP‐2 was positively correlated with FA of the subregion 2 (*r* = .557, *p* = .011) and negatively correlated with RD of the subregion 2 (*r* = −.499, *p* = .025). CPT‐IP‐3 was negatively correlated with RD of the subregion 1 (*r* = −.554, *p* = .011) and subregion 2 (*r* = −.492, *p* = .027), and positively correlated with FA of the subregion 2 (*r* = .540, *p* = .014). CPT‐IP‐4 was negatively correlated with RD of the subregion 1 (*r* = −.565, *p* = .009) and subregion 2 (*r* = −.480, *p* = .032), and positively correlated with FA of the subregion 2 (*r* = .510, *p* = .022). No significant associations of the callosal integrity changes with AHI, Mean SaO_2_, Nadir SaO_2_, ESS, and TBPM were found.

## 4 DISCUSSION

In the present study, we jointly used tractography and multiple diffusion metrics to investigate microstructural integrity alterations of the corpus callosum subregions in OSA. In comparison with healthy controls, OSA patients exhibited decreased FA and increased MD and RD in the anterior CC (subregions 1 and 2). In addition, we observed that worse prospective memory and sustained attention were associated with lower microstructural integrity of the anterior CC in OSA patients.

Corpus callosum damage has been consistently reported in OSA patients (Algin, Akin, Ocakoglu, & Ozmen, 2016; Kumar et al., 2012, 2014; Macey et al., 2008, 2012; Xiong et al., 2017) as well as in mice exposed to long‐term intermittent hypoxia (Veasey et al., 2013). We found that the affected CC subregions in OSA were located in the anterior rather than posterior part, which is consistent with some previous whole‐brain DTI studies demonstrating white matter integrity changes in the anterior CC in this disorder (Kumar et al., 2012, 2014; Macey et al., 2008). One possible explanation is the different myelination sequence and fiber composition in distinct CC subregions. CC is part of the highest order‐latest maturing neural network of the brain (Pujol, Vendrell, Junque, Marti‐Vilalta, & Capdevila, 1993). The fibers of CC are fully established before birth, and experience‐dependent pruning and elimination of fibers are more than balanced by a rostral‐caudal pattern of myelination that increases callosal size and lasts into young adulthood (Giedd et al., 1996). This rostral‐caudal myelination sequence indicates that the CC subregions might have distinct time windows of high vulnerability to negative experience. In regard to fiber composition, the anterior CC has the highest concentration of small, unmyelinated axons in the structure (Aboitiz, Scheibel, Fisher, & Zaidel, 1992). Combined, we speculate that the earlier myelination and distinctive fiber composition of the anterior CC may render it more vulnerable to OSA. However, in light of a trend toward reduced FA of the subregion 5 (Table S3) and previous findings of abnormal CC splenium microstructure in OSA patients (Xiong et al., 2017), we cannot rule out the possibility that the nonsignificant group differences in diffusion metrics of the posterior CC may result from the relatively small sample size.

In OSA patients, poorer performances in prospective memory and sustained attention were found to be associated with worse microstructural integrity of the anterior CC connecting bilateral prefrontal cortex and bilateral premotor and supplementary motor cortex. Prospective memory is a multiphase process including intention encoding, maintenance, and retrieval (Cona, Scarpazza, Sartori, Moscovitch, & Bisiacchi, 2015). This cognitive function implicates a complex neural network including the prefrontal cortex (Cona et al., 2015; Gonneaud et al., 2014) and premotor and supplementary motor cortex (Cona et al., 2015; Hashimoto, Umeda, & Kojima, 2011). Similarly, sustained attention is a multicomponent mental process involving distributed brain regions including the prefrontal cortex and premotor and supplementary motor cortex (Fortenbaugh, DeGutis, & Esterman, 2017; Langner & Eickhoff, 2013). Based on these prior findings, one may speculate that aberrant interhemispheric communications between bilateral prefrontal cortex and bilateral premotor and supplementary motor cortex, resulting from abnormal fiber connections, may contribute to the dysfunctions of prospective memory and sustained attention in OSA patients. However, although OSA‐related impairments in sustained attention (Karimi et al., 2015; Luz et al., 2016; Simoes, Padilla, Bezerra, & Schmidt, 2018) and prospective memory (Zhang et al., 2017) have be previously reported, no significant intergroup differences in these cognitive domains were observed in this study, which may result from the possibility of a type II error due to the small sample size. Of note, the finding of significant correlations between altered diffusion metrics and cognitive performances exhibiting no intergroup differences highlights the multifaceted and complex nature of these relationships, which need to be clarified in a larger sample.

White matter integrity changes in the anterior CC are mainly characterized by decreased FA and increased MD and RD. FA is a comprehensive reflection of the water diffusion profile, and its reduction can be determined by both parallel and perpendicular diffusivity. MD increase observed in our patient sample is in contrast to the previous findings of either increased or decreased MD in OSA (Kumar et al., 2012; Xiong et al., 2017). This discrepancy could perhaps be explained by clinical heterogeneity of patient samples, such as variation in disease stages. MD procedures can be used to distinguish acute from chronic stages after hypoxia, with reduced values in the acute stage, values similar to those of normal conditions in the subacute stage, and increased values in the chronic stage (Ahlhelm, Schneider, Backens, Reith, & Hagen, 2002). In the acute stage after hypoxemia, cytotoxic edema reduces extracellular water and leads to cell and axonal swelling, resulting in restricted water diffusion that contributes to reduced MD values. In the subacute stage, cytotoxic and vasogenic edemas co‐occur. Cytotoxic edema leads to a reduction in MD values as a consequence of by increased tissue barriers, whereas vasogenic edema leads to an increase in MD values, which results in MD values either little‐changed or similar to those of control conditions. In the chronic stage, demyelination or axonal loss will reduce tissue barriers, increase extracellular volume, and escalate vasogenic edema, all of which will lead to increased MD values. The current observation of increased MD suggests that most of the enrolled patients may be in the chronic stage of OSA‐induced hypoxemia. Moreover, increased RD and unchanged AD in this study indicate that the neuropathological mechanism underlying OSA‐related CC damage might be myelin disruption or loss rather than axonal injury (Beaulieu, 2002; Song et al., 2005; Trip et al., 2006), which supports the notion that myelin is more vulnerable to hypoxia than axons in OSA (Kumar et al., 2014).

There are several limitations in this study. First, we did not correct for multiple comparisons and many of the effects would not survive this correction because of limited statistical power due to the relatively small sample size. Second, the healthy controls did not undergo overnight PSG examinations. Thus, there was a lack of reliable objective evidence for an absence of OSA in our controls, although they were screened by a detailed interview to ensure an absence of symptoms including chronic snoring or nocturnal apneas. Third, extensive work in humans and animals has shown that obesity may lead to microstructural changes in the CC (Kullmann et al., 2016; Sherman et al., 2016). In this study, higher BMI was found in OSA patients relative to healthy controls. Although BMI was considered a covariate of no interest in the analyses, we cannot eliminate its potential effect completely. Finally, only patients with moderate‐to‐severe OSA were included in this study, which precludes us from generalizing our findings to the total population with OSA. Future studies are warranted to test whether patients with mild OSA also show similar integrity alterations in the CC subregions.

In conclusion, we examined white matter integrity changes in the CC at the subregional level in OSA by employing a combination of DTI tractography and multiple diffusion metrics. We found that OSA patients exhibited microstructural disruptions in the anterior CC, which were associated with worse prospective memory and sustained attention. These findings indicate that the selective impairments of the anterior CC may help clarify the neural substrates of cognitive impairments in OSA.

## CONFLICT OF INTEREST

The authors declare no conflict of interests.

## AUTHOR CONTRIBUTIONS

Jiajia Zhu and Yongqiang Yu designed this study and Biao Zhang wrote the draft of the manuscript. Dao‐min Zhu and Yu Zhang undertook psychiatric and psychometric assessments. Jiajia Zhu performed image processing and statistical analyses. Biao Zhang and Wenming Zhao operated the magnetic resonance imaging (MRI) machine. Ying Yang and Cun Zhang managed literature searches. All authors contributed to and have approved the final manuscript.

## Data Availability

The data that support the findings of this study are available on request from the corresponding author. The data are not publicly available due to privacy or ethical restrictions.
