# Supplementary material for: Selective microstructural integrity impairments of the anterior corpus callosum are associated with cognitive deficits in obstructive sleep apnea
Source: Brain Behav. 2019 Nov 20;9(12):e01482. doi: 10.1002/brb3.1482 (PMC6908858; doi:10.1002/brb3.1482)
Supplement: Supplementary file 1 [file BRB3-9-e01482-s001.docx]

Supplementary Materials

**Table S1．**Inter-rater reliability for diffusion metrics of the corpus callosum.

| Region |  | FA | |  | MD | |  | AD | |  | RD | |
| --- | --- | --- | --- | --- | --- | --- | --- | --- | --- | --- | --- | --- |
|  |  | *ICC* | *P* |  | *ICC* | *P* |  | *ICC* | *P* |  | *ICC* | *P* |
| Subregion 1 |  | 0.997 | <0.001 |  | 0.998 | <0.001 |  | 0.996 | <0.001 |  | 0.998 | <0.001 |
| Subregion 2 |  | 0.998 | <0.001 |  | 0.983 | <0.001 |  | 0.965 | <0.001 |  | 0.993 | <0.001 |
| Subregion 3 |  | 0.978 | <0.001 |  | 0.933 | <0.001 |  | 0.857 | <0.001 |  | 0.962 | <0.001 |
| Subregion 4 |  | 0.963 | <0.001 |  | 0.905 | <0.001 |  | 0.885 | <0.001 |  | 0.927 | <0.001 |
| Subregion 5 |  | 0.985 | <0.001 |  | 0.997 | <0.001 |  | 0.994 | <0.001 |  | 0.993 | <0.001 |
| Whole CC |  | 1.000 | <0.001 |  | 0.999 | <0.001 |  | 0.999 | <0.001 |  | 1.000 | <0.001 |

Abbreviations: ICC, intra-class correlation coefficient; FA, fractional anisotropy; AD, axial diffusivity; MD, mean diffusivity; RD, radial diffusivity; CC, corpus callosum.

Table S2．Diffusion metrics of the corpus callosum.

| Region |  | FA | |  | MD | |  | AD | |  | RD | |
| --- | --- | --- | --- | --- | --- | --- | --- | --- | --- | --- | --- | --- |
|  |  | PT | HC |  | PT | HC |  | PT | HC |  | PT | HC |
| Subregion 1 |  | 0.54±0.03 | 0.54±0.03 |  | 8.07±0.30 | 7.91±0.28 |  | 13.71±0.47 | 13.50±0.39 |  | 5.24±0.36 | 5.12±0.36* |
| Subregion 2 |  | 0.52±0.03 | 0.53±0.02* |  | 8.07±0.25 | 7.91±0.30* |  | 13.44±0.30 | 13.34±0.33 |  | 5.38±0.36 | 5.19±0.35* |
| Subregion 3 |  | 0.55±0.02 | 0.55±0.03 |  | 8.11±0.26 | 8.15±0.33 |  | 13.87±0.28 | 13.96±0.32 |  | 5.22±0.33 | 5.24±0.40 |
| Subregion 4 |  | 0.53±0.30 | 0.53±0.27 |  | 8.39±0.39 | 8.38±0.45 |  | 14.13±0.42 | 14.06±0.51 |  | 5.52±0.47 | 5.54±0.48 |
| Subregion 5 |  | 0.58±0.02 | 0.58±0.02 |  | 8.24±0.31 | 8.18±0.21 |  | 14.52±0.43 | 14.46±0.38 |  | 5.11±0.31 | 5.03±0.23 |
| Whole CC |  | 0.55±0.02 | 0.56±0.02 |  | 8.15±0.25 | 8.05±0.22 |  | 14.02±0.31 | 13.91±0.29 |  | 5.21±0.30 | 5.12±0.27 |

**P* < 0.05.

The data are presented as the mean ± standard deviation. For illustration, all values of AD, RD and MD were multiplied by 1000. Abbreviations: CC, corpus callosum; FA, Fractional Anisotropy; AD, axial diffusivity; RD, radial diffusivity; MD, Mean diffusivity; HC, healthy controls; PT, patients with obstructive sleep apnea

Table S3．Inter-group differences in diffusion metrics of the corpus callosum.

| Region |  | FA | |  | AD | |  | MD | |  | RD | |
| --- | --- | --- | --- | --- | --- | --- | --- | --- | --- | --- | --- | --- |
|  |  | *F* | *P* |  | *F* | *P* |  | *F* | *P* |  | *F* | *P* |
| Subregion 1 |  | 3.705 | 0.061 |  | 4.023 | 0.051 |  | 0.442 | 0.510 |  | 4.518 | 0.040* |
| Subregion 2 |  | 4.882 | 0.033* |  | 5.042 | 0.030* |  | 0.372 | 0.546 |  | 5.742 | 0.021* |
| Subregion 3 |  | 2.102 | 0.155 |  | 0.211 | 0.649 |  | 0.704 | 0.406 |  | 0.993 | 0.325 |
| Subregion 4 |  | 0.583 | 0.449 |  | 0.793 | 0.378 |  | 0.449 | 0.507 |  | 0.739 | 0.395 |
| Subregion 5 |  | 3.790 | 0.058 |  | 0.373 | 0.545 |  | 0.326 | 0.571 |  | 1.733 | 0.195 |
| Whole CC |  | 3.634 | 0.064 |  | 2.316 | 0.136 |  | 0.073 | 0.789 |  | 3.300 | 0.077 |

**P* < 0.05.

Abbreviations: FA, fractional anisotropy; AD, axial diffusivity; RD, radial diffusivity; MD, mean diffusivity; CC, corpus callosum.
